# Annual acknowledgement of manuscript reviewers

**DOI:** 10.1186/1742-4755-11-9

**Published:** 2014-02-13

**Authors:** José M Belizán, Natasha Salaria

**Affiliations:** 1 Institute for Clinical Effectiveness and Health Policy (IECS), Buenos Aires, Argentina; 2 BioMed Central Limited, UK

## Abstract

**Contributing Reviewers:**

The editor of *Reproductive Health* would like to extend a sincere thank you to all our valued reviewers listed below who contributed to the journal in Volume 10 (2013). Without the co-operation and collaboration of peer reviewers, these manuscripts would remain static in a submission system with high quality articles unpublished and inaccessible to the public on a global scale. We are very pleased to have a diverse range of reviewers from high, middle and low income countries, contributing to the dissemination of research findings in an open access journal. Please accept our deepest thanks for your knowledge, time and continuing efforts.

## 

Clara Aarts

Sweden

Karim Abawi

Switzerland

Mohammad Jalal Abbasi-Shavazi

Iran

Muhammad Ahmed Abdullah

Pakistan

Sileshi Abeya

Ethiopia

Farina Abrejo

Pakistan

Asri Adisasmita

Indonesia

Mesganaw Fantahun Afework

Ethiopia

Sohail Agha

United States of America

Mohammed Ali

Australia

Niloufer Ali

Pakistan

Gretchen Antelman

United States of America

Isabella Archie

United Kingdom

Nega Assefa

Ethiopia

Berhanemeskel Atsbeha

Ethiopia

Tariq Azim

Ethiopia

Syed Khurram Azmat

Pakistan

Frank Baiden

Ghana

Babagana Bako

Nigeria

Sibhatu Biadgilign

Ethiopia

Jessica Bird

Canada

Paul Blumenthal

United States of America

Maria Bortolini

Brazil

Doortje Braeken

United Kingdom

Sally Brown

United Kingdom

Lynn Callister

United States of America

Claire Carson

United Kingdom

Lidia Casas

Chile

Laura Castleman

United States of America

Chandra-Mouli Venkatraman

Switzerland

Charoenchai Chiamchanya

Thailand

John Cleland

United Kingdom

Tim Colbourn

United Kingdom

Mercedes Colomar

Uruguay

Mark Connolly

Switzerland

Gabriela Cormick

Argentina

Divanise Correia

Brazil

Phyllis Dako-Gyeke

Ghana

Jess Davis

Australia

Joshua Davis

United States of America

Adriane Delicio

Brazil

Thérèse Delvaux

Benin

Donna Denno

United States of America

Kebede Deribe

Ethiopia

Richard Derman

United States of America

Madhu Dixit Devkota

Nepal

John Ditekemena Congo

Democratic Republic

Somé Donmozoun Télesphore

Burkina Faso

Catherine Egbe

South Africa

Omaima El Gibaly

Egypt

Sebastian Eliason

Ghana

Laila El-Zeini

Egypt

Cyril Engmann

United States of America

Gedefaw Abeje Fekadu

Ethiopia

Herve Fernandez

France

Marcelo Ferreira

Brazil

Jane Fisher

Australia

Britt Friberg

Sweden

Enrique Gadow

Argentina

Cynthia Geary

United States of America

Tesfay Gebregzabher Gebrehiwot

Ethiopia

Terefe Gelibo

Ethiopia

Yoseph Gessesse

Ethiopia

Demian Glujovsky

Argentina

Azita Goshtasebi

Iran

Rena Greifinger

United States of America

Alexis Guedes

Brazil

Carlos Güida

Chile

Yan Guo

China

Bernard Guyer

United States of America

Gwyn Hainsworth

United States of America

John Hall

Australia

Claudia Hanson

Sweden

Miriam Hartmann

United States of America

Fariha Haseen

Bangladesh

Robert Henry

United States of America

Ellen Hodnett

Canada

Susie Hoffman

United States of America

Justus Hofmeyr

South Africa

Laili Irani

United States of America

Eliana Jimenez-Soto

Australia

Neetu John

India

Sarah Johnson

United Kingdom

Stan Kachnowski

United States of America

Ros Kane

United Kingdom

Ravneet Kaur

India

Method Kazaura

Tanzania

James Kiarie

Kenya

Joyce Kinaro

Kenya

Marion Koso-Thomas

United States of America

Fiona Kouyoumdjian

Canada

Ramesh Kumar

Pakistan

Yihunie Lakew

Ethiopia

Gloria Langat

United Kingdom

Malinee Laopaiboon

Thailand

Elena Lebetkin

United States of America

Tippawan Liabsuetrakul

Thailand

Fernando Luiz Affonso Fonseca

Brazil

Pisake Lumbiganon

Thailand

Mercedes Mac Mullen

Argentina

Shawn Malarcher

United States of America

Alice March

United States of America

Francisco Mardones

Chile

Pamela Martey

Ghana

Anthony Massinde

Tanzania

Matthews Mathai

Switzerland

Maureen Mayhew

Canada

Arslan Mazhar

Pakistan

Michael Mbizvo

Switzerland

Anthony Mbonye

Uganda

Alexandre Megale

Brazil

Wubegzier Mekonnen

Ethiopia

Legese Alemayehu Mekuria

Ethiopia

Tesfaye Mengistu

Ethiopia

Ana Paula Pierre Moraes

Brazil

Rafael Moroni

Brazil

Elise Murowchick

United States of America

Declare Mushi

Tanzania

Ruta Nadisauskiene

Lithuania

Katie Newby

United Kingdom

Gustavo Nigenda

Mexico

Béatrice Nikiema

Canada

Peter Norrie

United Kingdom

Adeline Nyamathi

United States of America

Anicet Nzabonimpa

Rwanda

Francis Obare

Kenya

Ekechi Okereke

Nigeria

Bolajoko O. Olusanya

Nigeria

Kwaku Oppong Asante

Ghana

Lucia O'Sullivan

Canada

Kola Oyediran

Nigeria

Rodolfo Pacagnella

Brazil

Nitika Pant Pai

Canada

Omrana Pasha

Pakistan

Andrea Barnabas Pembe

Tanzania

Nanlesta Pilgrim

United States of America

Hammad Qazi

Canada

Susan Qua

Canada

Parvin Rahnama

Iran

Michael Ripple

United States of America

Katrina A. Ronis

Pakistan

Katrina Aminah Ronis

Pakistan

Sara Rosenbaum

United States of America

Anne Rossier Markus

United States of America

Mariano Salazar

Sweden

Sarah Saleem

Pakistan

Solita Sarwono

Netherlands

Rachel Savage

United Kingdom

Lisa Schubert

Australia

Agumasie Semahegn

Ethiopia

Babar Tasneem Shaikh

Pakistan

Techalew Shimelis

Ethiopia

Kavita Singh

United States of America

Gita Sinha

United States of America

Jeffrey Michael Smith

United States of America

Lucy Smith Paintain

United Kingdom

Adam Sonfield

United States of America

Joao Paulo Souza

Switzerland

William Story

United States of America

John Stover

United States of America

Elizabeth Sutherland

United States of America

Tara Symonds

United Kingdom

Charlotte Tawiah-Agyemang

Ghana

Charles Teller

United States of America

Dessalegn Tesso

Ethiopia

Nai-peng Tey

Malaysia

Tizta Tilahun

Ethiopia

Burcu Tokuc

Turkey

Ozge Tuncalp

Switzerland

Joshua Vogel

Switzerland

Fernando Volpe

Brazil

Jennifer Wagman

United States of America

Joyce Wamoyi

Tanzania

Bobby Webster

United States of America

Sharon Weir

United States of America

Amanda Whitten

Canada

Carrie Williams

United Kingdom

Recep Yildizhan

Turkey

